# Expression of Toll-Like Receptor 2 in Glomerular Endothelial Cells and Promotion of Diabetic Nephropathy by *Porphyromonas gingivalis* Lipopolysaccharide

**DOI:** 10.1371/journal.pone.0097165

**Published:** 2014-05-16

**Authors:** Yoshihiko Sawa, Shunsuke Takata, Yuji Hatakeyama, Hiroyuki Ishikawa, Eichi Tsuruga

**Affiliations:** 1 Department of Morphological Biology, Fukuoka Dental College, Fukuoka, Japan; 2 Department of Oral Growth & Development, Fukuoka Dental College, Fukuoka, Japan; SRI International, United States of America

## Abstract

The toll-like receptor (TLR) has been suggested as a candidate cause for diabetic nephropathy. Recently, we have reported the TLR4 expression in diabetic mouse glomerular endothelium. The study here investigates the effects of the periodontal pathogen *Porphyromonas gingivalis* lipopolysaccharide (LPS) which is a ligand for TLR2 and TLR4 in diabetic nephropathy. In laser-scanning microscopy of glomeruli of streptozotocin- and a high fat diet feed-induced type I and type II diabetic mice, TLR2 localized on the glomerular endothelium and proximal tubule epithelium. The TLR2 mRNA was detected in diabetic mouse glomeruli by *in situ* hybridization and in real-time PCR of the renal cortex, the TLR2 mRNA amounts were larger in diabetic mice than in non-diabetic mice. All diabetic mice subjected to repeated LPS administrations died within the survival period of all of the diabetic mice not administered LPS and of all of the non-diabetic LPS-administered mice. The LPS administration promoted the production of urinary protein, the accumulation of type I collagen in the glomeruli, and the increases in IL-6, TNF-α, and TGF-β in the renal cortex of the glomeruli of the diabetic mice. It is thought that blood TLR ligands like *Porphyromonas gingivalis* LPS induce the glomerular endothelium to produce cytokines which aid glomerulosclerosis. Periodontitis may promote diabetic nephropathy.

## Introduction

Diabetes mellitus is a dysfunction of the glucose metabolism caused by an absence or insufficient production of insulin and it is classified into type 1 with autoimmune destruction of insulin-secreting β cells, and type 2: metabolic disorders which comprise 95% of diabetes mellitus cases. A hyperglycemic environment generates advanced glycation end products (AGE) like Nε-(carboxymethyl) lysine (CML) and the hydroxyl radical produced in the process damage endothelial cells due to long-term oxidative stress, further, the recognition of blood AGE through AGE receptors induces the production of various cytokines from endothelial cells and leukocytes, resulting in cardiovascular complications [1], [2], [3]. Diabetic nephropathy is a very serious complication of chronic renal disease, characterized by renal failure or dysfunction with podocyte loss, glomerular basement membrane thickening, tubular dysfunction, and expansion of the mesangium which is composed of extracellular matrix proteins from mesangial cells around glomerular capillaries. Diabetic nephropathy causes occlusion of glomerular capillaries based on glomerulosclerosis and arises from excess accumulation of extracellular matrix but details of the mechanisms are not fully elucidated [4], [5], [6], [7].

Recently, the toll-like receptor (TLR) has been suggested as a candidate cause of diabetic nephropathy [8], [9], [10]. The TLRs which are sensors for pathogen-associated molecular patterns common to bacterial components also recognize extracellular matrix degradation products, fatty acids, and AGE, and the recognition finally results in cytokine production in leukocytes and blood endothelial cells [11], [12], [13], [14], [15]. Basically, gram-positive bacterial components like lipoteichoic acid are recognized by TLR2 and gram-negative bacterial components like lipopolysaccharide (LPS) are recognized by TLR4, and further LPS from the periodontal pathogen *Porphyromonas gingivalis* binds both TLR2 and TLR4 [16], [17], [18]. A hyperglycemic environment of at least 11.2 mM glucose, which is the diagnostic reference value for diabetes mellitus, and the presence of CML and free fatty acid elevate TLR2 and TLR4 levels in monocytes, glomeruli, and proximal tubules in diabetic nephropathy, and in the cultured rat renal proximal tubular cell line NRK-52E [19], [20], [21]. The TLR ligand engagement gives rise to production of pro-inflammatory cytokines like tumor necrosis factor-α (TNF-α) and interleukin (IL)-6, and also production of anti-inflammatory cytokines like transforming growth factor-β (TGF-β), and the cytokines finally promote an overproduction of mesangial matrix, such as type I collagen [12], [13], [22], [23], [24]. However, there are no reports that have shown the TLR localization and the ligand-induced cytokine production in diabetic renal constituents by high-resolution optical images. Recently, we have reported TLR4 expression in glomerular endothelial cells in type 1 and type 2 diabetic mice by confocal laser scanning microscopy [25].

Blood components morphologically easily accumulate in the tufts of glomerular-shaped capillaries in high blood pressure environments and influence glomerular endothelial cells. It is thought that the recognition of blood AGE through the receptors on glomerular endothelial cells induces TLR expression, and that the ligand engagement with TLR triggers the cytokine production of glomerular endothelium, which promotes diabetic nephropathy.

The present study aimed to examine the TLR2 expression in glomerular endothelial cells in diabetic mice and the effect of LPS from the periodontal pathogen *Porphyromonas gingivalis* on the progress of diabetic nephropathy.

## Materials and Methods

Two animal studies were performed to achieve the project goals. Study 1: A streptozotocin (STZ) and a genetic background induced diabetic mouse model were used to investigate the localization of TLR2 in the diabetic renal constituents. Study 2: The survival rate with *Porphyromonas gingivalis* LPS administration was compared for normal and diabetic mice to assess the promotion of diabetic nephropathy by the interaction of TLR and oral pathogens. The studies here used control and experimental groups with eight mice in each group (2/cage). The manuscript was prepared following the ARRIVE guidelines.

### Animals

The experimental protocol for the animal use was reviewed and approved by the Animal Experiment Committee of Fukuoka Dental College in accordance with the principles of the Helsinki Declaration. Breeding and experiments were performed in a room with a 100% controlled atmosphere which had passed an examination for bacteria and is located in the Fukuoka Dental College Animal Center. Mice grew normally and lived healthily under conventional atmosphere conditions with normal feeding in cages and rooms in which temperature (22°C) and humidity (55%) were completely controlled. The mice were housed with an inverse 12 hour day-night cycle with lights on from 7:00pm.

Humane endpoints were used in the experiments as a rapid and accurate method for assessing the health status of the mice, that is, mice with loss of the ability to ambulate (inability to access food or water) were euthanized by induction anesthesia (1 l/min of 2% isoflurane mixed with 30% oxygen and 70% nitrous oxide with an anaesthetic apparatus) followed by cervical dislocation and intraperitoneal injections with sodium pentobarbital (10 ml/kg, Nembutal, Abbott Laboratories, North Chicago, IL).

The streptozotocin (STZ)-injected closed line ICR mice (Japan Clea Inc., Osaka, Japan) and KK/TaJcl (KK/Ta; Japan Clea) mice with a genetic background of insulin-independent diabetes were used as the diabetic models [25]. Five-week-old male ICR mice were given a single intraperitoneal injection of STZ (200 mg/kg body weight)(Sigma, St. Louis) in a 0.05 M citric acid buffer at pH 4.5 (20 mg/ml). The non-diabetic controls were ICR mice intraperitoneally administered with 0.05 M citric acid buffer only. The injection was conducted under inhalation anesthesia with 2% isoflurane. Carprofen (7.5 mg/kg; Wako Pure Chemical Industries, Ltd., Osaka, Japan) was used pre-injection for preemptive analgesia and post-injection every 12–24 hours. The animal condition was checked every 8 hours for the first 2 days after the injection, and every 12 hours thereafter. The blood glucose was checked once a week after the injection by measurements of the blood glucose using a Glutest Sensor (Sanwa Kagaku Kenkyusyo CO., LTD., Nagoya, Japan). One week after the STZ injection diabetes was confirmed and ICR mice with blood glucose above 600 mg/dl for four months were designated as STZ-induced type I diabetic mice. Nine-week-old male KK/Ta mice were fed a high fat diet feed (HFD32 [HF], Japan Clea) for five months, and KK/Ta mice with blood glucose levels above 600 mg/dl were designated as HF-induced type II diabetic mice. The non-diabetic controls were KK/Ta mice given normal feed. All mice were euthanized by induction anesthesia with 2% Isoflurane followed by cervical dislocation at the end of the designated period of the experiments, and the mouse tissue was collected.

### LPS Administration

To investigate the survival rate of the diabetic mice with LPS, *Porphyromonas gingivalis* LPS (3 mg/kg, LD50 = 30 mg/kg body weight)(Wako) was administered repeatedly intraperitoneally once a week to the STZ-induced diabetic mice which had shown blood glucose levels above 600 mg/dl for four months [26], [27]. The injection was conducted under inhalation anesthesia with 2% isoflurane. Carprofen (7.5 mg/kg; Wako) was used pre-injection for preemptive analgesia and post-injection every 12–24 hours. The animal condition was checked every 8 hours for the first 2 days after the injection, and every 12 hours thereafter. The urine of the ICR mice, the ICR mice with repeated LPS administrations, the STZ-induced diabetic mice, and the STZ-induced type I diabetic mice with repeated LPS administrations, was analyzed for sugar, protein, and bleeding by urine reagent strips (Uriace, Terumo Corporation, Tokyo, Japan) once a week from the 1st LPS administration and at humane endpoints described above. All mice were euthanized by induction anesthesia with 2% Isoflurane followed by cervical dislocation at the end of the designated period of the experiments, and the mouse tissue was collected.

### Immunohistochemistry

The mouse monocyte-macrophage cell line J774A.1 (JCRB9108, JCRB Cell Bank, Osaka, Japan) and human leukemia cell line HL60 (JCRB0085) were cultured in RPMI1640 medium with L-glutamine and 10% fetal bovine serum, and seeded onto slide glass. Frozen human kidney tissue sections of type II diabetes mellitus (BioChain Instisute, Inc., Newark, CA) were used for the immunohistochemical examinations. Cells and frozen 10 µm mouse kidney tissue sections cut in a cryostat were placed on the slide glass. The cells and sections were fixed in 100% methanol for 5 min at −20°C, treated with 0.1% goat serum for 30 min at 20°C, and then treated for 8 hrs at 4°C with PBS containing 0.1% goat serum and the following primary antibodies (1 µg/ml): hamster monoclonal anti-mouse podoplanin (AngioBio Co., Del Mar, CA) as a podocyte marker, rat monoclonal anti-mouse TLR2 (R&D Systems Inc., Minneapolis, MN), rabbit polyclonal anti-mouse vascular endothelial (VE)-cadherin (Abcam plc., Cambridge, UK), rabbit polyclonal anti-mouse platelet endothelial cell adhesion molecule-1 (PECAM-1, Abcam), rabbit polyclonal anti-mouse IL-6 (Abcam), rabbit polyclonal anti-mouse TNF-α (Abcam), rabbit polyclonal anti-mouse TGF-β (Abcam), and rabbit polyclonal anti-type I collagen (Abcam). After the treatment with primary antibodies the sections were washed three times in PBS for 10 min and immunostained for 0.5 hr at 20°C with 0.1 µg/ml of second antibodies: Alexa Fluor (AF) 488 or 568-conjugated goat anti-hamster, goat anti-rabbit, or goat anti-rat IgGs (Probes Invitrogen Com., Eugene, OR). The immunostained sections were mounted in 50% polyvinylpyrrolidone solution and examined by fluorescence microscopy (BZ-8100, Keyence Corp., Osaka, Japan) or confocal laser-scanning microscopy (LSM710, Carl Zeiss, Jena, Germany) with an ×63 oil Plan Apochromatic objective lens (numerical aperture ×1.3).

### *In situ* Hybridization

The frozen 10 µm sections cut in a cryostat and placed on slide glass were fixed in 4% paraformaldehyde-PBS and air dried in an oven at 60°C for 1 hr. All procedures of the *in situ* hybridization for mouse TLR2 mRNA and 3,3′-Diaminobenzidine (DAB) staining on the hybridized genes were performed by the RNAscope 2.0 FFPE Assay (Advanced Cell Diagnostics, Inc., Hayward, CA) and a probe for mouse TLR2 mRNA (NM_011905.3, 1069–2039, Advanced Cell Diagnostics) including negative and positive controls according to the manufacturer’s instructions. Several negative controls (e.g. sense probe and no probe) were run in parallel with the experimental reactions.

### Reverse Transcription (RT)-PCR and Real-time PCR

The tissue was peeled away from the kidney within a 5 mm square by an 18-gauge needle under a stereoscopic microscope. The total RNA extraction from the tissue was performed with a QIAshredder column and an RNeasy kit (Qiagen, Inc., Tokyo, Japan). Contaminating genomic DNA was removed using DNAfree (Ambion, Huntingdon, UK), and the RT was performed on 30 ng of total RNA, followed by 30 cycles of PCR for amplification using the Ex Taq hot start version (Takara Bio Inc., Otsu, Japan) with 50 pM of primer sets for mouse β-actin, TLR2, IL-6, TGF-β, and TNF-αmRNAs (Table 1), where the specificities had been confirmed by the manufacturer (Sigma-Genosys Ltd., Cambridge, UK). The RT-PCR products were separated on 2% agarose gel (NuSieve; FMC, Rockland, ME, USA) and visualized by Syber Green (Takara). The correct size of the amplified PCR products was confirmed by gel electrophoresis and amplification of accurate targets was confirmed by sequence analysis. To quantify mRNA generation, cDNA samples were analyzed by real-time quantitative PCR. A total of 1 µl of cDNA was amplified in a 25-µl volume of PowerSYBR Green PCR Master Mix (Applied Biosystems, Foster City, CA, USA) in a Stratagene Mx3000P real-time PCR system (Agilent Technologies, Inc., Santa Clara, CA, USA), and the fluorescence was monitored at each cycle. Cycle parameters were 95°C for 15 min to activate Taq followed by 35 cycles of 95°C for 15 s, 58°C for 1 min, and 72°C for 1 min. For the real-time analysis, two standard curves were constructed from amplicons for both the β-actin and target genes in three serial 4-fold dilutions of cDNA. The β-actin or target gene cDNA levels in each sample were quantified against β-actin or the target gene standard curves by allowing the Mx3000P software to accurately determine each cDNA unit. Finally, target gene cDNA units in each sample were normalized toβ-actin cDNA units. Finally, the relative target gene expression units were expressed as arbitrary units, calculated according to the following formula: relative target gene expression units = target gene cDNA units/β-actin cDNA units.

**Table 1:** Sequence of primers.

protein	bp	upper (5′–3′)	lower (5′–3′)
β-actin	411	GTTCTACAAATGTGGCTGAGGA	ATTGGTCTCAAGTCAGTGTACAG
podoplanin	192	CACCTCAGCAACCTCAGAC	AAGACGCCAACTATGATTCCAA
IL-6	263	ATGTTCTCTGGGAAATCGTGGAAAT	TCTCTGAAGGACTCTGGCTTTGT
TGF-β	372	GCGTGCTAATGGTGGACCG	CGTGGAGTTTGTTATCTTTGCTGTC
TNF-α	355	GCGAGGACAGCAAGGGACT	GAGGCCATTTGGGAACTTCTCAT

### Measurements of the Immunostained Areas of Tissue Sections

Areas immunostained by anti-type I collagen and anti-podoplanin were measured around different glomeruli (10/section) in laser-scanned microscopic images at 819× magnification by ImageJ (National Institutes of Health, Bethesda, MD). The relative volume of type I collagen accumulation was expressed by the mean of the ratio: area of type I collagen in a glomerulus/area of a glomerulus within podoplanin-positive podocytes.

### Statistics

All experiments were repeated five times, and data are expressed as mean+SD. The statistical significance of differences (p<0.05) was determined by the Student’s t test and one-way ANOVA with STATVIEW 4.51 software (Abacus concepts, Calabasas, CA, USA).

## Results

### Distribution of TLR2-positive Cells in the Diabetic Mouse Kidney

**Figure 1:**
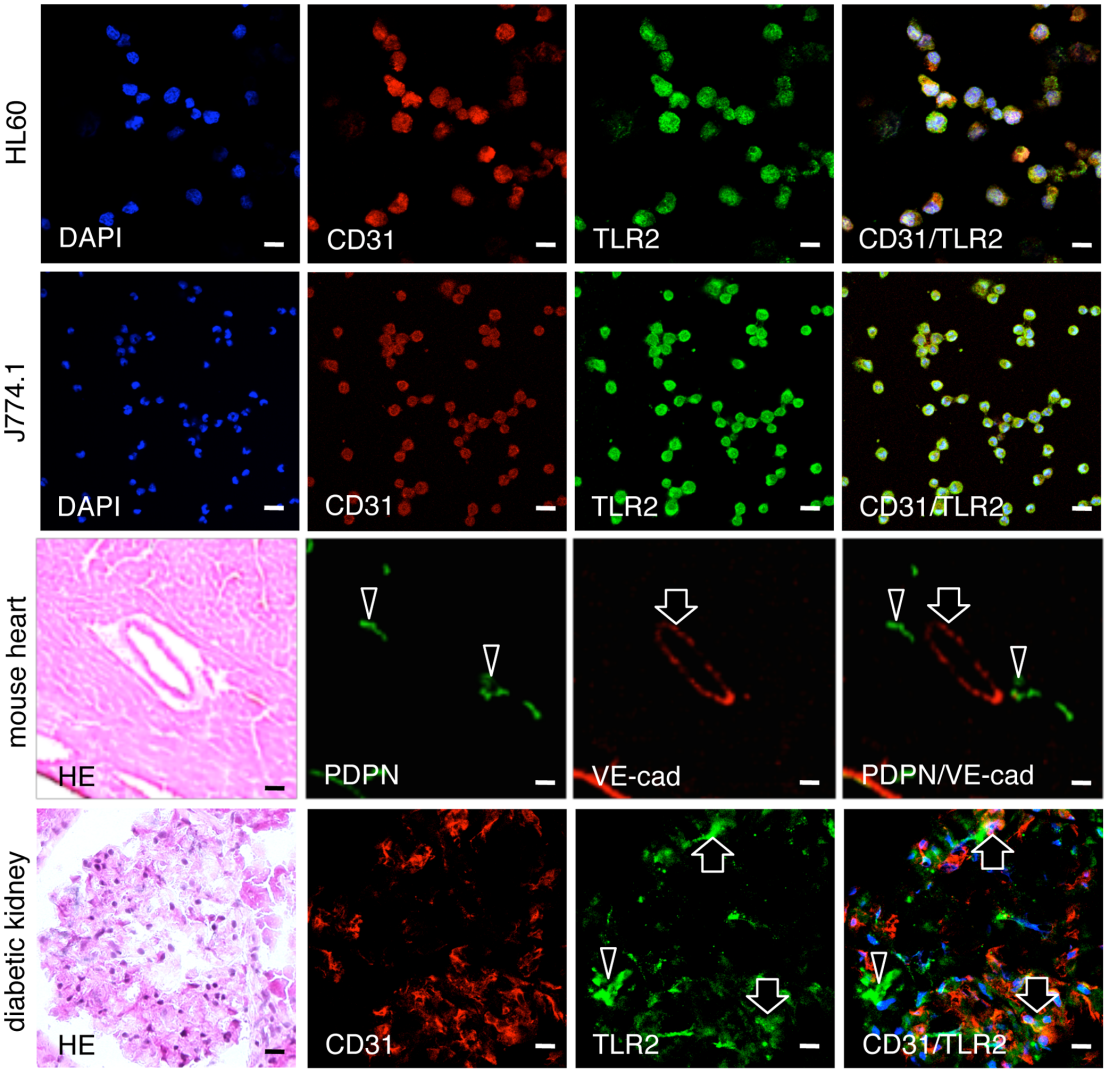
Immunostaining for TLR2, PECAM-1, podoplanin, and VE-cadherin. Mouse and human leukocyte cell line J774A.1 and HL60 were immunostained with both anti-PECAM-1 (CD31) and anti-TLR2, and nuclei were stained by DAPI. In the mouse heart section, lymphatic vessels (arrowheads) were immunostained by anti-podoplanin (PDPN) and a blood vessel (arrows) was stained by anti-VE-cadherin (VE-cad). In the human kidney section with type II diabetic nephropathy, there are glomerular endothelial cells immunostained with both anti-PECAM-1 (CD31) and anti-TLR2 (arrows), and cells that are only immunostained with anti-TLR2 (arrowheads). Bar: 20 µm.

**Figure 2:**
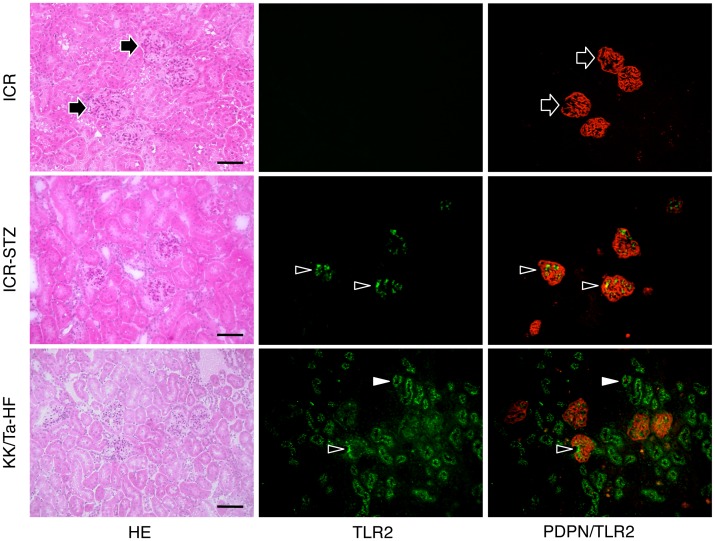
Distribution of TLR2-positive cells in diabetic mouse kidneys. The immunostained sections were re-stained by HE staining. The HE staining shows that renal tubules are expanded in the cortex of the kidneys of STZ-induced type I diabetic ICR mice (ICR-STZ) and of HF-induced type II diabetic KK/Ta mice (KK/Ta-HF). In the non-diabetic ICR mouse kidney section, all of the glomeruli were immunostained with the antibody for the podocyte marker, podoplanin (PDPN, arrows, staining red), while there were no cells reacting with anti-TLR2. In the ICR-STZ and KK/Ta-HF mouse kidney sections, there are areas immunostained by anti-TLR2 (arrowheads, staining green) in all of the podoplanin-positive glomeruli. In the KK/Ta mouse kidney sections, podoplanin-negative proximal tubules which are more strongly stained with eosin than the distal tubules are also immunostained by anti-TLR2 (white arrowheads, staining green). In the ICR-STZ and KK/Ta-HF mouse kidney sections, distal tubules, collecting tubules, and blood vessels outside glomeruli are not stained. Bar: 100 µm.

**Figure 3:**
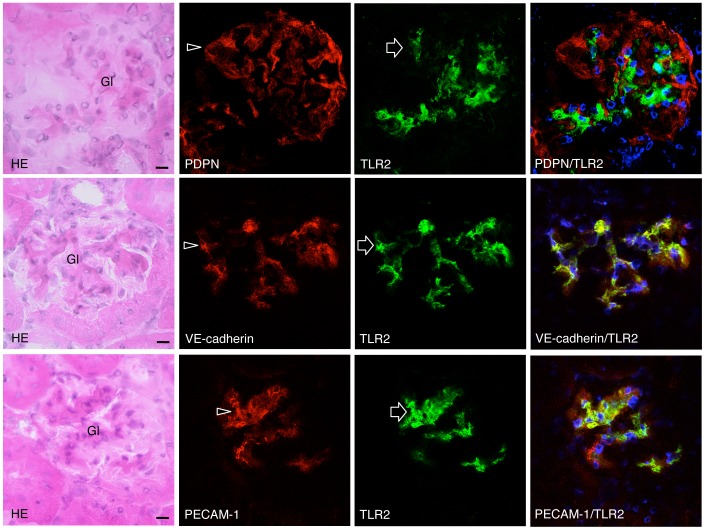
Localization of TLR2 in the glomeruli of STZ-induced type I diabetic ICR mouse kidneys. The HE staining showed that glomeruli (Gl) of the diabetic mice are subject to sclerosis. In the laser-scanning confocal microscopy, the region reacting with anti-podoplanin (PDPN, arrowhead) does not coincide with the region reacting with anti-TLR2 (arrows) in the merged image while the regions reacting with VE-cadherin and anti-PECAM-1 (arrowheads) coincide with the regions reacting with anti-TLR2 (arrows) in the merged images (rightmost column). Bar: 20 µm.

**Figure 4:**
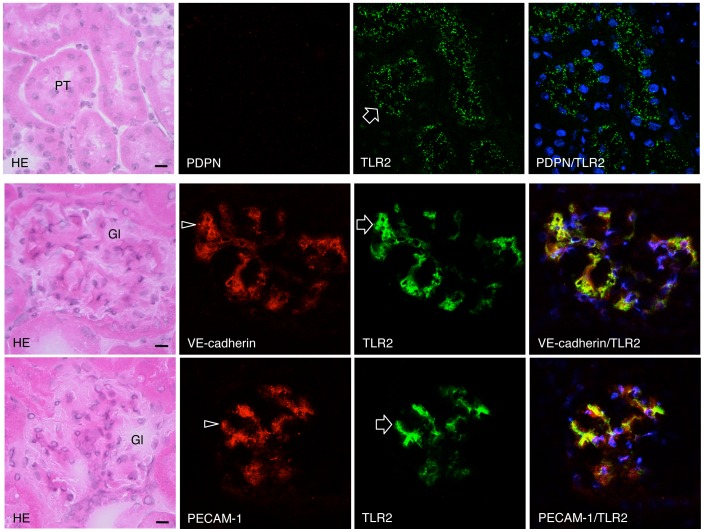
Localization of TLR2 in the glomeruli of HF-induced type II diabetic KK/Ta mouse kidneys. The HE staining showed that glomeruli (Gl) of the KK/Ta-HF mice are subject to sclerosis. In the laser-scanning confocal microscopy, epithelial cells of proximal tubules (PT) did not react with anti-podoplanin (PDPN) but reacted with anti-TLR2 (arrows) at the inside. The regions reacting with VE-cadherin and anti-PECAM-1 (arrowheads) coincided with the regions reacting with anti-TLR2 (arrows) in the merged images. Bar: 20 µm.

The mouse monocyte-macrophage cell line J774A.1 and human leukocyte cell line HL60 were immunostained with both anti-PECAM-1 and anti-TLR2 (Fig. 1). In the mouse heart, lymphatic vessels and blood vessels were immunostained by anti-podoplanin and anti-VE-cadherin, respectively. There were no cross reactions with primary or secondary antibodies in any of the negative controls and there were no differences in the fluorescence intensity of the target proteins of the single and double immunostaining with primary antibodies (not shown). In the human kidney samples with diabetic nephropathy, there were PECAM-1-positive glomerular endothelial cells reacted with both anti-PECAM-1 and anti-TLR2, and cells only immunostained with anti-TLR2. The TLR2 expression was immunohistochemically examined in the renal constituents of STZ-induced type I and HF-induced type II diabetic mice (Fig. 2). In the renal cortex of diabetic mice, glomeruli showed sclerosis based on the mesangial thickening and renal tubules were expanded, and the tissue is collapsed by edema. There were no glomeruli immunostained by anti-TLR2 in the kidney tissue of non-diabetic control mice untreated with STZ (Fig. 2) or HF (not shown), whereas almost all glomeruli had areas immunostained by anti-TLR2 in the kidney tissue of STZ- and HF-induced diabetic mice. In the kidney tissue of HF-induced diabetic mice, there were proximal tubules immunostained by anti-TLR2, while distal tubules, collecting tubules, and blood vessels outside the glomeruli were not stained (Fig. 2). In the laser-scanning confocal microscopy on the glomeruli of the STZ- and HF-induced diabetic mice, podoplanin-positive podocytes were not immunostained with anti-TLR2 while VE-cadherin and PECAM-1-positive glomerular endothelial cells were stained (Fig. 3, Fig. 4), and reaction products with anti-TLR2 were present at the inside of proximal tubule epithelial cells of HF-induced diabetic mice.

### Gene Expression of TLR2 in the Diabetic Mouse Kidney

**Figure 5:**
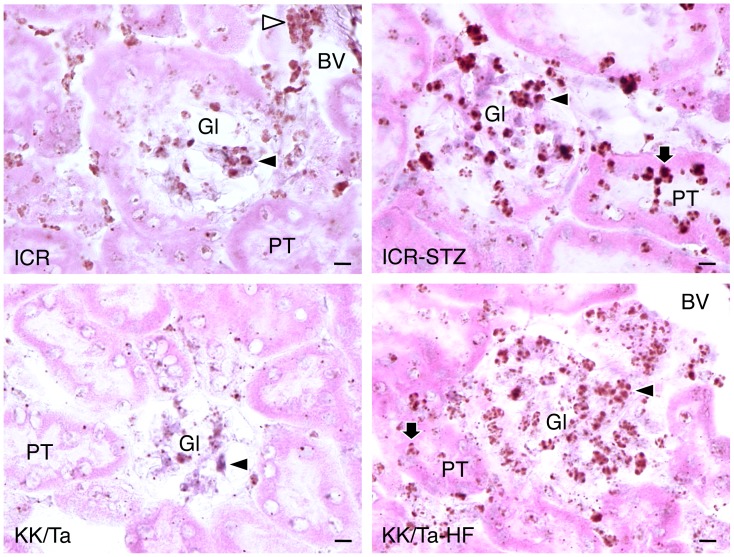
*In situ* hybridization for TLR2 mRNA of the diabetic mouse kidneys. DAB signals for the genes hybridized with the probe for TLR2 mRNA (closed arrowheads) are present weakly at the glomeruli (Gl) of non-diabetic ICR and KK/Ta mice, and strongly at the glomeruli of STZ-induced type I diabetic ICR mice (ICR-STZ) and HF-induced type II diabetic KK/Ta mice (KK/Ta-HF). DAB signals are also positive at the proximal tubules (PT) of ICR-STZ and KK/Ta-HF. There are leukocytes with DAB signals on the internal peroxidase (open arrowheads) in the blood vessels (BV) of the non-diabetic ICR mouse section (top left). Bar: 20 µm.

**Figure 6:**
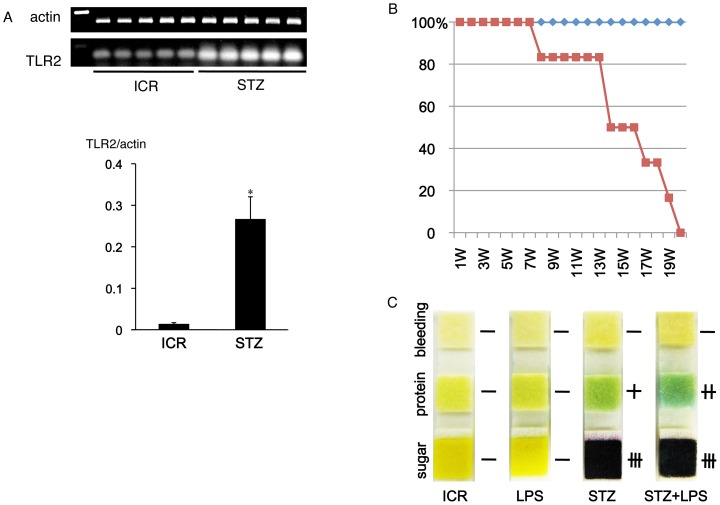
Quantitative analysis for TLR2 gene expression in the diabetic mouse kidneys and the effects of *Porphyromonas gingivalis* LPS on diabetic nephropathy. (A) Tissue PCR for TLR2 mRNA of the mouse renal cortex. The RT-PCR and real-time PCR analysis show that the mRNA amounts were larger in the STZ-induced type I diabetic ICR mice (STZ) than in the non-diabetic ICR mice (ICR). *Significantly different (p<0.05) (B) Survival curve of the STZ-induced type I diabetic ICR mice with repeated intraperitoneal administrations of *Porphyromonas gingivalis* LPS. All of the 6 diabetic mice with LPS (red curve) died within the survival period of all of the diabetic mice without LPS and the non-diabetic mice with LPS (blue curve). (C) Urinalysis for STZ-induced type I diabetic ICR mice with LPS at the 8th week of survival. Urine reagent strips show sugar, protein, and bleeding in the mouse urine of non-diabetic ICR mice (ICR), non-diabetic ICR mice with LPS (LPS), STZ-induced type I diabetic ICR mice without LPS (STZ), and the diabetic mice with LPS (STZ+LPS). Extensive amounts of urinary sugar was observed in the diabetic mice and the diabetic mice with LPS, and the urinary protein level was higher in the diabetic mice with LPS than in the diabetic mice without LPS.

*In situ* hybridization for TLR2 mRNA showed that almost all signals on the genes hybridized with the probe for TLR2 mRNA were present in glomeruli but there were only few in the constituents outside the glomeruli of non-diabetic mouse kidney tissue (Fig. 5). In the STZ-induced type I and HF-induced type II diabetic mouse kidney tissue, the signals for TLR2 mRNA were present at glomeruli and proximal tubules to a stronger extent than in the kidneys of non-diabetic mice. Analysis of RT-PCR and quantitative real-time PCR for TLR2 mRNA in the mouse renal cortex showed that the mRNA amounts were larger in STZ-induced diabetic mice than in the non-diabetic mice (Fig. 6A).

### Effects of *Porphyromonas gingivalis* LPS on Diabetic Nephropathy

**Figure 7:**
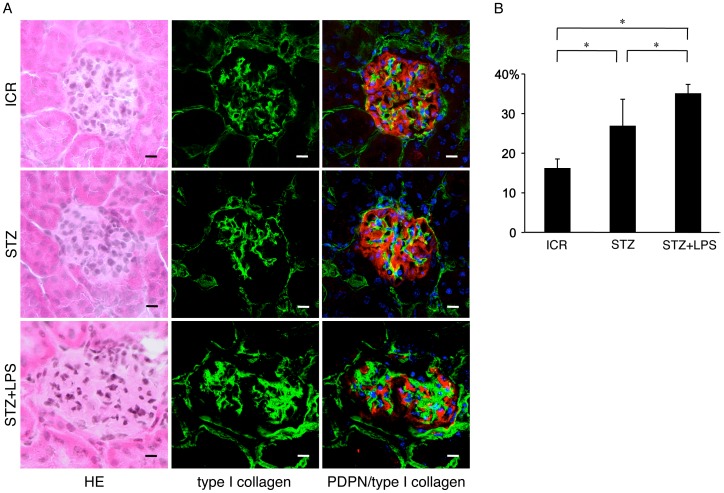
Progress of collagen accumulation in the kidneys of diabetic mice repeatedly administered *Porphyromonas gingivalis* LPS. (A) Immunohistochemistry for the expression of type I collagen and podoplanin (PDPN, red) on glomeruli by laser-scanning confocal microscopy. The intensity of the immunostaining for type I collagen is stronger in the glomeruli of STZ-induced type I diabetic ICR mice with the repeated intraperitoneal administrations of *Porphyromonas gingivalis* LPS (STZ+LPS) than in the diabetic mice without LPS (STZ) and in the non-diabetic ICR mice (ICR). Bar, 20 µm. (B) Quantitative analysis for type I collagen positive areas in the glomeruli. Areas immunostained by anti-type I collagen and anti-podoplanin in ten different glomeruli of laser-scanning microscopic images were measured by ImageJ. The relative volume of type I collagen accumulation in the glomeruli was expressed by a mean ratio: area of type I collagen in a glomerulus/area of a glomerulus within podoplanin-positive podocytes. There were statistically significant differences in the relative accumulations of type I collagen in the glomeruli of non-diabetic ICR mice (ICR) and STZ-induced type I diabetic ICR mice (STZ), and between the diabetic mice and the diabetic mice with LPS (STZ+LPS). Data are expressed as means+SD. *Significantly different (*p*<0.01).

All of the STZ-induced type I diabetic mice subjected to repeated intraperitoneal administration of *Porphyromonas gingivalis* LPS died within the survival period of all of the diabetic mice not administered LPS and non-diabetic mice administered LPS (Fig. 6B). Urinalysis show substantial concentrations of urinary sugar in the STZ-induced diabetic mice both with and without repeated LPS administrations, and that urinary protein levels were higher in the diabetic mice with LPS administration than in the diabetic mice without LPS administration (Fig. 6C). The intensity and area of reaction products with anti-type I collagen were stronger in the glomeruli of STZ-induced diabetic mice with LPS administration than in diabetic mice without LPS administration or in non-diabetic mice (Fig. 7A). There were statistically significant differences in the relative volumes of type I collagen accumulation in the glomeruli of non-diabetic mice and STZ-induced diabetic mice, and between the diabetic mice and the diabetic mice with LPS administration (Fig. 7B).

### Cytokine Induction by *Porphyromonas gingivalis* LPS in the Diabetic Mouse Kidney

**Figure 8:**
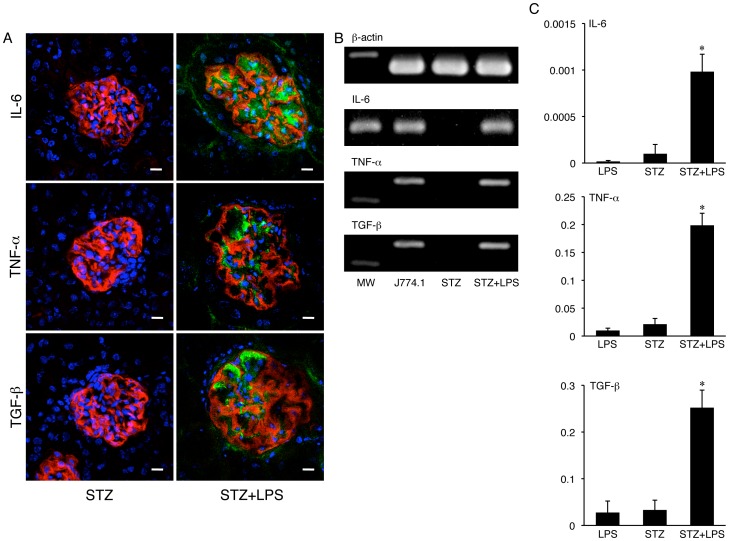
Cytokine induction by *Porphyromonas gingivalis* LPS in the diabetic mouse kidneys. (A) Immunohistochemistry for the expression of cytokines and podoplanin (PDPN) in glomeruli by laser-scanning confocal microscopy. The IL-6, TNF-α, and TGF-β were detected in the glomeruli of STZ-induced type I diabetic ICR mice with the repeated intraperitoneal administration of *Porphyromonas gingivalis* LPS (STZ+LPS) whereas the no cytokines were detected in the kidneys of the diabetic mice without LPS (STZ). Bar: 20 µm. (B) Tissue RT-PCR for cytokine mRNAs in mouse kidneys. The amplicons of IL-6, TNF-α, and TGF-β mRNAs were detected from both the renal cortex of STZ-induced type I diabetic ICR mice with the repeated intraperitoneal administrations of *Porphyromonas gingivalis* LPS (STZ+LPS) and cultured mouse macrophage J774.1 with LPS, whereas they were not detected from the diabetic mice without LPS (STZ). (C) Tissue real-time PCR for cytokine mRNAs in mouse kidneys. The gene expression amounts of IL-6, TNF-α, and TGF-β were statistically significantly larger in the kidney tissue of STZ-induced type I diabetic ICR mice with the repeated intraperitoneal administrations of *Porphyromonas gingivalis* LPS (STZ+LPS) than in the diabetic mice without LPS (STZ) and in the non-diabetic ICR mice with LPS (LPS). Data are expressed as means+SD. *Significantly different (*p*<0.01).

The IL-6, TNF-α, and TGF-β were immunohistochemically detected in the glomeruli of STZ-induced type I diabetic mice repeatedly administered intraperitoneal *Porphyromonas gingivalis* LPS whereas no cytokines were detected in the kidneys of the diabetic mice without LPS (Fig. 8A) or in the non-diabetic mice with LPS (not shown). The expressions of IL-6, TNF-α, and TGF-β mRNAs in the renal cortex were detected in the STZ-induced diabetic mice with LPS administration but not in the diabetic mice without LPS (Fig. 8B) or non-diabetic mice with LPS (not shown). In tissue real-time PCR for cytokine mRNAs in the renal cortex, the mRNA amounts of IL-6, TNF-α, and TGF-β in the renal cortex were statistically significantly larger in the STZ-induced diabetic mice with LPS than in the diabetic mice without LPS or the non-diabetic mice with LPS (Fig. 8C).

## Discussion

### Expression of TLR2 in Glomerular Endothelial Cells of Diabetic Mice

Monocyte-macrophage cell lineages commonly express innate immune receptor TLR2 and leukocyte adhesion molecule PECAM-1. Blood endothelial cells express PECAM-1 and adherence junction molecule VE-cadherin, and lymphatic endothelial cells express the podocyte marker podoplanin [12], [25]. These were immunohistochemically confirmed here in the reactions of the antibodies to cultured cells and tissue sections with no cross reactions, indicating that the study here was successful (Fig. 1). There are renal cells expressing TLR2 in the human kidney with diabetic nephropathy and TLR2 expression was detected in almost all glomeruli of the type I and type II diabetic mouse kidneys here which showed glomerulosclerosis and edema, and detected in the type II diabetic mouse proximal tubules but not in the non-diabetic mouse kidneys (Fig. 1,2). These results suggest that there are TLR2-expressing cells in the glomeruli and proximal tubules under the condition of diabetic nephropathy. In the laser-scanning confocal microscopy on the glomeruli of type I and type II diabetic mice, the TLR2-expressing area coincided with the VE-cadherin and PECAM-1-double positive areas, suggesting that the TLR2-expressing cells in the glomeruli with diabetic nephropathy were mainly glomerular endothelial cells (Fig. 3, 4). The reaction products with anti-TLR2 were also localized at the inside of the diabetic mouse proximal tubules, and it is thought that proximal tubule epithelial cells have the ability to express TLR2 on the luminal side under the condition of diabetic nephropathy. Signals for TLR2 mRNA were detected in the glomeruli and proximal tubules but only weakly in the constituents outside the glomeruli by the *in situ* hybridization, and the signals were stronger in the diabetic mice than in the non-diabetic mice (Fig. 5). The TLR2 mRNA amounts were larger in diabetic mice than in non-diabetic mice as determined by tissue RT-PCR of the renal cortex (Fig. 6A). These suggest that the enhancement of TLR mRNA expression occurs in the glomeruli and proximal tubules under diabetic conditions. The TLR2 induction has been reported in both diabetic patients and experimentally induced diabetes. The TLR2 up-regulates in monocytes under hyperglycemia in type I and type II diabetic patients, and the TLR2 expression is enhanced in patients with diabetic nephropathy [9], [10], [17], [19], [28], [29]. Glucose exposure induces the TLR expression and transforming growth factor-β production in mouse mesangial cells, and TLR knockout mice show less progress of diabetic nephropathy than wild-type diabetic mice [30], [31]. The study here showed the localized expressions of TLR2 on the glomerular endothelial cells and proximal tubule epithelial cells, whereas the Bowman capsules, collecting tubules, and renal lymphatic and blood vessels outside glomeruli did not express TLR2 in type I and type II diabetic mouse kidneys. Further, we have recently showed the localized expression of TLR4 on the glomerular endothelial cells but not in other renal constituents in type I and type II diabetic mouse kidneys by confocal laser-scanning microscopy [25]. It is thought that glomerular endothelium plays the principal role with TLR2 and 4 in glomerulosclerosis. Since glomerular capillaries easily accumulate blood components in the winding loops of capillaries like a ball of thread, it is one of the target organs of type III hypersensitivity. It is thought that blood AGEs which accumulate in glomeruli and effuse into the proximal tubules may induce TLR2 expression by recognition through AGE receptors on glomerular endothelial cells and proximal tubule epithelial cells.

### *Porphyromonas gingivalis* LPS Effects Promotion of Diabetic Nephropathy

The recognition of TLR ligands induces cytokine production in TLR expressing cells [11], [12], [13]. Crude LPS from the periodontal pathogen *Porphyromonas gingivalis* activates both TLR2 and TLR4 because of the covalently bound lipopeptide [16], [17], [18]. In diabetic mice which have elevated urinary sugar excretion, urinary protein excretion increased by repeated administrations of *Porphyromonas gingivalis* LPS and all of the LPS-administered mice died within the survival period of all of the LPS-untreated diabetic mice and non-diabetic mice with LPS, suggesting that *Porphyromonas gingivalis* LPS hastens the mortality risk due to diabetes mellitus (Fig. 6B). The relative volume of type I collagen accumulation in the glomeruli was larger in diabetic mice which were treated with repeated administrations of *Porphyromonas gingivalis* LPS than in LPS-untreated diabetic mice, suggesting that *Porphyromonas gingivalis* LPS promotes type I collagen accumulation (Fig. 7). Further, more IL-6, TNF-α, and TGF-β were produced in the glomeruli of diabetic mice which were treated with repeated administrations of *Porphyromonas gingivalis* LPS than in the glomeruli of LPS-untreated diabetic mice or LPS-administered non-diabetic mice (Fig. 8). These results suggest that the production of IL-6, TNF-α, and TGF-β occurred due to the repeated administrations of LPS where the administration of LPS does not cause the induction of these three under healthy conditions. All the components derived from gastrointestinal bacteria are metabolized in the liver while microorganisms from infection lesions of the oral cavity and pharynx directly enter the kidney through the systemic circulation. Severe periodontitis easily gives rise to bacteremia, and it is thought that blood TLR ligands like LPS or LTA, which originate from periodontal pathogen microorganisms, accumulate in the glomeluri, and that TLR ligands induce leukocytes and glomerular endothelial cells to produce proinflammatory cytokine IL-6 and TNF-α which enhance tissue destruction, and anti-inflammatory cytokine like TGF-β, which allows mesangial cells around glomerular endothelial cells to produce extracellular matrix proteins playing a central role in tissue repair and glomerulosclerosis [32], [33], [34], [35], [36], [37]. The TLR ligands originating from periodontitis like *Porphyromonas gingivalis* LPS may promote diabetic nephropathy.
